# A Hospital Function at the Mansion House

**Published:** 1912-06-15

**Authors:** 


### A HOSPITAL FUNCTION AT THE MANSION HOUSE.

### The Dinner in Aid of St. Bartholomew's Hospital.

With the object of liquidating a debt on St. Bartholomew's Hospital due to the bankers, amounting to £57,000, a distinguished company of some 170 gentlemen dined together in the Egyptian Hall of the Mansion House on Wednesday evening, June 5, at the invitation of the Right Hon. the Lord Mayor (Sir Thomas Boor Crosby, M.D.). Among those present were, besides the Lord Mayor, the Right Hon. Lord Sandhurst, G.C.S.I., G.C.I.E. (Treasurer of St. Bartholomew's Hospital), the Right Hon. Lord Aldenham, the Right Hon. Lord Blyth, the Right Hon. and the Right Rev. the Lord Bishop of London, the Right Hon. Lord Hollenden, Sir Thomas Barlow, Bart., K.C.V.O., F.R.S. (President of the Royal College of Physicians), Sir William Church, Bart., K.C.B., Sir Alfred Cripps, K.C.V.O., K.C., M.P., Sir John Henry Luscombe, J.P. (Chairman of Lloyd's), Sir Henry Burdett, K.C.B., K.C.V.O., Sir Ernest Flower, Sir Lauder Brunton, Bart., F.R.S., Dr. Robert Jones, F.R.C.P., F.R.C.S., Sir William Soulsby, C.B., C.I.E., Dr. Samuel West, F.R.C.P., Dr. Norman Moore, F.R.C.P., Sir Anthony Bowlby, C.M.G., F.R.C.S., Mr. C. B. Lockwood, F.R.C.S., Mr. Bruce Clarke, F.R.C.S., Mr. Edwin J. Layton (Hon. Secretary Appeal Committee), Mr. Thomas Hayes (Clerk to the Governors of St. Bartholomew's Hospital).

### The Toasts.

The Lord Mayor proposed the toasts "The King" and "The Queen, Queen Alexandra, the Prince of Wales, and the other Members of the Royal Family" in brief but felicitous phrases, and they were musically pledged.

#### St. Bartholomew's Hospital.

In submitting the toast of the evening, "St. Bartholomew's Hospital," the Lord Mayor said the company were met in order to support a hospital which, for many hundreds of years, had existed on the spot it still occupied for the great work of supplying medical and surgical needs to poor sufferers who were unable to afford to purchase them. The hospital now needed support from the public, and from his Lordship's knowledge of his fellow-citizens he felt confident that the support required would be cheerfully given in answer to the appeal being made by the Treasurer and the Almoners. Whenever he had advocated the dispensation of charity the very word "hospital" had been quite enough to originate help, for it meant a recognition that there was no shamming in illness, and that broken legs undeniably meant suffering, and that, however undeserving a person might be in the ordinary way, the fact that he was ill placed him within the sphere of sympathy. From no fault of administration, but simply owing to excess of expenditure over income, and largely in order to provide increased accommodation, the hospital needed assistance, not only temporarily, but more or less permanently. He reminded the gathering that St. Bartholomew's was the only general hospital within the City boundaries, and its position was a very important one. For nearly a thousand years the hospital had helped the sick poor, bringing multitudes back to health and strength and enabling them again to earn their livelihood. And the medical instruction side of a hospital's work should never be lost sight of, for without that the utility of any large hospital would be divided. But he was not appealing for any medical school; the appeal was being made to carry on and extend the remedial work, to pay for the greatly increased accommodation provided there for the patients, and he had confidence that the public would rise to the occasion. He coupled the toast with the name of the Right Hon. Lord Sandhurst, the Treasurer.

#### The Right Hon. Lord Sandhurst.

The Treasurer responded to the toast at considerable length. He said that in former days, when he was chief administrator at Middlesex Hospital, he frequently had to respond for the needs of that institution, but never before, to his knowledge, had it been necessary to ask the citizens of London to assemble at a dinner in aid of St. Bartholomew's Hospital. It was very fitting as well as hospitable of the Lord Mayor to permit the present dinner in aid of the only general hospital within the City boundaries to take place at the Mansion House. (Applause.) There was abundant proof that the promoters of a good cause never appealed to the Mansion House in vain, and it was a satisfaction to him that the present occupant of the Chief Magistracy of the City should be a member of the learned and gentle medical profession.

When, three and a half years ago, he became Treasurer of St. Bartholomew's Hospital, he made it his duty to investigate the state of the hospital's finances, and to rummage into every branch of its administration, to see whether those who were qualified to judge thought that the institution was being properly and economically managed, and for this purpose he assembled a small committee of gentlemen accustomed to business, some of whom had businesses of their own. These gentlemen were Mr. Stone (Almoner), Mr. Acton Davis (Almoner), Mr. Matthew Wallace (of the Common Council), and Sir William Church (then President of the College of Physicians). That committee, after looking into every corner of the hospital's work, concluded that the institution was well managed. He laid stress upon that, because when an establishment got into deep water there were plenty of people ready to say—and sometimes it could be said with truth—that there must be or have been bad management when it was well known that the revenues were so large. But—and this applied to individuals as to institutions—there might be large revenues, but there might also be very large mortgages, which considerably depleted the resources. But he wished to point out to the present gathering, and beyond it, that management was one thing and policy quite another thing. Twelve years ago it was deliberately decided to buy 1½ acre of private hospital land—a very expensive proceeding, involving, as it did, a sum of more than £250,000. He was careful always to avoid criticising the action of his predecessors, but it was obvious that when laying out such a large sum as that one must sell the stock, which was interest-bearing, in order to pay the bill; and it was also necessary to borrow money in other directions to make up the difference. Therefore the hospital now found itself in the position of having a deficit of about £7,000 or £8,000 a year, whereas fifteen or twenty years ago the hospital was doing its work at a profit of £2,000 or £3,000 a year. The building had cost a great deal of money, and though the public contributed a large sum for that purpose, including the Pathological block, an amount exceeding that by £14,000 had been spent. With regard to the Pathological block, medical men would bear him out when he said it was not a luxury, for it was of no use to have a hospital of 600 or 700 beds unless it was up to date in regard to the latest skill and knowledge.

### The Suggested Removal.

Ten years ago an idea was started that it would be wise to remove St. Bartholomew's Hospital into the country, to sell the land on which it now stands, and to buy land elsewhere. That attracted considerable attention at the time, and a committee sat to consider the matter, under the chairmanship of Sir Marcus Samuel, and he (the speaker) was a member of that committee. The result of the opinions and evidence invited was that the hospital should remain where it was. That he regarded as a wise decision, especially remembering the vast number who came by rail to receive the hospital's benefits. Of course, in every large institution difficulties were to be encountered, and since the Unification of Parishes Act the rates had naturally increased. Some said, "How can you expect me to subscribe to your hospital? Taxation has gone up so much, and into the bargain there is an Insurance Act which provides for this business which you aspire to carry on." He admitted there was something in those observations. But his friends present would agree with him that the hospitals should be maintained as charities, rather than that they should become State hospitals, however well they might be likely to be managed. But the efforts of St. Bartholomew's Governors were not merely directed to providing a certain amount of income; he also had the great ambition that, in course of time, a proper home would be provided for the hospital nurses. (Applause.)

### Who have Given the Money.

The hospital's appeal had done very well, although competition in charitable matters had been severe, and the usual factors had been added to recently by the appeal connected with the terrible tragedy of the *Titanic*. No one would begrudge the response which had been made on that account, but it naturally made a difference to the subscriptions to ordinary charities. His Lordship then proceeded to mention the gifts of various great corporations to the fund, including the Mercers' Company 1,000 guineas, the Goldsmiths' Company 1,000 guineas, the Grocers' Company £1,000, which were in direct response to the appeal issued by the Lord Mayor. Mr. John Smithers had already raised a sum exceeding £1,700 on the Stock Exchange. Sir John Luscombe had sent in a cheque for £1,800 from a collection at Lloyd's. Lord Hollenden had sent in a list from the textile trades amounting to over £1,200. Mr. Arthur Hill, chairman of the Finance Committee, had done a great deal of work, but as the donations came in direct to the hospital one could not quite measure his influence. Mr. Acton Davis, who had so long served as an almoner, had sent £1,000. Mr. Florence, another almoner, had sent from his private friends over £800. The Trustees of Smith's Charity had sent £1,000, Mr. Nivison sent £500, and Sir Julius Wernher sent £500. His friend Lord Cowdray had just returned from Mexico, so he communicated with him on the telephone, with the result that he gave £500. He also communicated with the owner of the winner of the Derby that day, and he promised him 50 guineas. (Applause.) The influential members of the Central Meat Market had been good enough to organise a substantial collection, which it was hoped would materially increase the hospital's annual revenue. Mr. John Hill had collected £346. He would not weary the gathering with a recital of the smaller amounts, but would like it to be known that in the autumn a theatrical performance would be arranged by Sir Ernest Flower, who had already done so much for the hospital. Many subscriptions of one guinea had been received, and they were highly esteemed.

### Mr. I'Anson's Retirement.

As showing the value of small things, he drew attention to a paragraph in a circular sent to members of the London Chamber of Commerce, in response to which £150 a year had already been received in one pound subscriptions. His Lordship spoke in high terms of the ungrudging work given by Mr. Layton in regard to the collections. The Governors had guaranteed for three years the sum of £4,612. The donations up to the time of the dinner were £16,120, and to that must be added the £4,612 of the Governors, and the grand total, including the amounts outstanding, was expected to reach £30,000. He contended that the public were well served by St. Bartholomew's Hospital, and he paid a tribute of praise to the staff for their disinterested work, which often entailed broken rest. And in those words of commendation he included the matron and the nurses, of whom it was impossible to speak too highly; he only wished they lived in a better house. He regretted that an old officer of the hospital had had to retire, after a great many years of service, on his pension, Mr. I'Anson. His work had been most valuable, as had also that of Mr. Hayes, the clerk to the Governors. He expressed his pleasure at seeing present Mr. I'Anson, the surveyor, who had been a zealous officer and a generous contributor to the funds. On behalf of the Governors and staff of the hospital he expressed gratitude to the Lord Mayor for his hospitality, and not only did he do so on behalf of those he had mentioned, but also in the name of the thousands which the hospital endeavoured to relieve.

### The Bishop of London's Speech.

The Bishop of London, in submitting the toast of "The Visiting Staff of the Hospital," said it was a fine tradition that men in the position of Lord Sandhurst should be found championing the cause of the hospitals, and it would be agreed that never was a more earnest appeal made on behalf of any hospital. An appeal on behalf of St. Bartholomew's Hospital must have a great attraction for any Bishop of London. One could not help admiring the cleverness of Lord Sandhurst in getting fifty guineas from the winner of the Derby. But he spoke mostly on behalf of those who, next to the doctors, knew the sick poor of London better than any other men—namely, the working parsons of London. For twenty-two years he laboured among the East End poor in that capacity, and he could not conceive what the clergyman's work among those poor would be without the hospitals. It was a great joy to see some denizen of the slums who had the misfortune to be ill or to meet with an accident being tended in clean and wholesome surroundings by cheerful and sympathetic doctors and nurses. He had many times preached at a stand in Victoria Park on a Hospital Sunday afternoon with a carpet spread around the stand, the condition being that if there were any standing around wearing top hats they were expected to give gold, and others silver and copper, according to their means, and had witnessed quite a copper shower, sometimes the collection amounting to £50. It indicated what the working men thought of their hospitals. And so great a reputation had St. Bartholomew's Hospital that there was a temptation for men feeling ill to fall by the way at a point near that hospital. Throughout the world "Bart.'s" had sent its graduates, and when he was feeling unwell at Luxor, in Egypt, it was a "Bart.'s" man who attended him. He spoke of the skill and attention given to every patient, the hospitals performing free for the poor operations for which others had often to pay 100 guineas. He coupled the toast with the names of two distinguished members of the staff, Dr. Samuel West and Mr. Bruce Clarke.

"Congratulations for a Colleague."

Dr. Samuel West, in responding to the toast, took the opportunity of congratulating a colleague upon holding the eminent position in the City of London which Sir Thomas Crosby occupied, the first member of the profession to do so. He also complimented him on the extraordinary energy and vitality which he exhibited in the discharge of his duties, an energy which must be envied by many younger men. He wondered whether even the Bishop of London had any idea that the toast he proposed included nearly 200 people, for there were forty members of the staff, fifty recognised medical officials not on the staff, and the army of students, whose work was an essential part of the life of the hospital, because the work could not go on without them. In drinking that toast they were pledging the efficiency and good working of the whole hospital. When St. Bartholomew's appealed for money, surprise was generally expressed because it was understood to be such a wealthy establishment. But Dr. West proceeded to show that that charge could not fairly be preferred. He was himself an optimist, and though the debt and the responsibilities were great at the present time, he did not believe that the hospital would be burdened very long with its large debt.

Economy in Bandages.

He showed that the extreme cleanliness, care, and orderliness observed at the hospital were really great factors in the cure of sickness, and that any small extra expense was more than justified. He applied the same line of reasoning to the question of diet and to the prolongation of the stay of patients in the hospital beyond what might be regarded as the minimum, for thereby the chances of a relapse were very greatly reduced. The tradition of the hospital was that everything should be of the best, and it was believed that in the end the best was the cheapest. Economy was carefully practised, but the best of all things cost money, though bought at contract prices. Such a small matter as having bandages five yards long as a routine length, instead of six yards, effected a saving of £180 a year. Some patients by their stay in hospital learned for the first time in their lives the value of cleanliness. And students were taught, at the most receptive part of their lives, how things ought to be done, and when they were scattered over the face of the globe they carried that practice with them. The history of the hospital in the past consisted of a long record of large resources carefully husbanded and economically administered, and, as required, wisely and generously spent. (Applause.)

Mr. Bruce Clarke also responded, and devoted his remarks largely to three factors in hospital work: the treatment of the patients, teaching of students, and the progress of medicine. He contended that it was impossible to teach adequately unless one was at the same time progressing, nor unless this progress existed could patients be treated properly. There was no keener critic than the student, and the continual questionings to which the teachers had to submit kept them on the alert. He detailed the case of a Crimean veteran and the way in which, though eighty years of age, he was successfully piloted through a series of illnesses and left the hospital in a very vigorous condition to celebrate his golden wedding.

The Livery Companies.

Dr. Norman Moore submitted this toast in a speech full of historical retrospect of an interesting nature connected with the great City corporations, from among which one would single out the crisis in the reign of King Henry VIII., when St. Bartholomew's Hospital was saved from destruction by the good offices of Sir Richard Gresham. The ultimate result of that distinguished citizen's work was that the King granted to the hospital a charter, that under which it had been administered up to the present time. He showed how generously the various corporations had supported the hospital throughout its long career, and expressed the cordial gratitude of the authorities of the institution. He had much pleasure in submitting the toast of "The Livery Companies," coupled with the name of Mr. H. Cullen.

Mr. H. Cullen, in responding, said that City Companies were often misunderstood, not in the City, but in other places. He had heard it said that they were close boroughs, of enormous wealth, which they selfishly and extravagantly spent. At all events, he hoped those Companies would do as much for charity and for education in the future as they had in the past.

The Visitors.

Sir Alfred Cripps, K.C.V.O., K.C., M.P., proposed the toast of "The Visitors," coupled with the name of Lord Aldenham. He echoed the wish that the hospitals would remain on their present voluntary basis, and confirmed the opinion that the needs of St. Bartholomew's had not arisen from either extravagant management or undue expenditure. The Bishop of London had well expressed the feelings which were entertained towards the hospital, and he (the speaker) wished to emphasise the great and self-denying work which men like Sir Thomas Barlow had done among the poor. In that connection also he mentioned with great satisfaction Mr. Sydney Holland's work at the London Hospital.

Lord Aldenham returned thanks in a brief speech, in which he expressed, on behalf of the visitors, much gratitude for their reception in the historic surroundings. He believed the Egyptian Hall had never before been utilised with greater pleasure to promote the cause of a great charity.

The Lord Mayor.

Lord Hollenden, in submitting this toast, spoke of the readiness with which the Lord Mayor agreed to allow the banquet to be held in the Mansion House, for which the cordial thanks of the hospital and the guests were due.

The Lord Mayor, in reply, expressed the Lady Mayoress' and his own thanks, and announced that the sum of £1,378, with £170 subscriptions, had been collected in the room, making a grand total of £33,000. He hoped that subscriptions would still continue to pour in, and the noble Treasurer desired that he would express thanks to Sir William Soulsby for all that he had done. He concluded in these words: "My Lords, gentlemen, members of my own profession, this will be a day memorable to me as long as I am spared, one of the pleasing recollections of this Mansion House at the end of my year of office as Chief Magistrate of this the greatest and the richest City the world has known." (Cheers.)

